# Characterization of the complete chloroplast genome sequence of *Potentilla gageodoensis* (rosaceae), endemic to the continental islands of Korea

**DOI:** 10.1080/23802359.2022.2067497

**Published:** 2022-04-21

**Authors:** Seon-Hee Kim, Ariun Shukhertei, JiYoung Yang, Seung-Chul Kim

**Affiliations:** a Department of Biological Sciences, Sungkyunkwan University, Suwon, Republic of Korea; b Research Institute for Dok-do and Ulleung-do Island, Kyungpook National University, Daegu, Republic of Korea

**Keywords:** Complete chloroplast genome, phylogenomic analysis, *Potentilla gageodoensis*, Rosaceae

## Abstract

This study reports the complete chloroplast genome sequence of a continental island endemic, *Potentilla gageodoensis*. The total plastome size was 156,397 bp, comprising one large single-copy (LSC; 85,768 bp), one small single-copy (SSC; 18,589 bp), and two inverted repeat (IR) regions (IR_a_ and IR_b_, each with 26,020 bp). The overall GC content was 36.92%, and the plastome contained 131 genes, comprising 84 protein-coding genes with two pseudogenes (*inf*A and *ycf*1), 37 transfer RNA genes, and eight ribosomal RNA genes. Phylogenetic analysis performed using 27 representative Rosoideae plastomes suggests that the genus *Potentilla* is not monophyletic and that *P. gageodoensis* is sister to the clade containing four taxa of *Potentilla* (*P. freyniana*, *P. freyniana* var. *chejuensis*, *P. stolonifera*, and *P. stolonifera* var. *quelpaertensis*). The present study reveals the taxonomic distinction of *P. gageodoensis* from its congeneric species in Korea and the plastome sequence obtained from this study can be used to study phylogenetic relationships and taxonomic status.

*Potentilla gageodoensis* M. Kim 2014, belonging to the subfamily Rosoideae of Rosaceae, is a perennial herb distributed on only a few continental islands of the Jeollanam-do Province, South Korea, in the southwestern part of the Korean Peninsula (So et al. 2014). It was originally described from a single island, Gageo-do Island, but was later found on Hong-do Island and a few uninhabited islands of South Korea. *Potentilla* L. is a large genus with approximately 400 species mainly distributed in the Northern Hemisphere (Persson et al. 2020). This genus has been known since ancient times, and numerous *Potentilla* species and their extracts have been widely used in different cultures (Tomczyk and Latté, 2009). Given the wide range of ploidy levels, from diploid (2x) to hexadecaploid (16x), polyploidization and hybridization have played important roles in the diversification of *Potentilla* (Kalkman, 2004; Dobes and Paule, 2010). Of the 17 *Potentilla* species known to occur in Korea, two taxa, *P. dickinsii* var. *glabrata* Nakai and *P. gageodoensis*, are endemic to the oceanic Ulleung Island and continental southern islands, respectively, whereas *P. squamosa* Sojak occurs in the central and southern Korean Peninsula (Lee, 2007; Heo, Lee et al. 2019). Morphologically, *P. gageodoensis* is closely related to *P. fragarioides* L., but it can be distinguished based on morphological characteristics including leaflet numbers, leaflet margins, and petal size (So et al. 2014). Little is known about the overall phylogenetic relationships among congeneric species of *Potentilla* in Korea as well as in East Asia. Plastome sequences of a few *Potentilla* species have recently been reported in Korea and China (Heo, Park et al. 2019; Yu et al. 2021). A lack of plastid genome sequences within the genus hinders our understanding of plastome organization and evolution, as well as the identification of useful plastid markers for DNA barcoding. In this study, we generated complete plastome sequences of *P. gageodoensis* and determined its phylogenetic position with respect to its congeneric species. This reference plastome of *P. gageodoensis* will be useful for future phylogenomic analyses of the genus, as well as for disentangling the complex evolutionary history of *Potentilla* as an effective organelle genome.

*P. gageodoensis* was sampled from an uninhabited island of Daesambu-do Islands (Jeollanam-do Province; N34 03.481 E127 23.71; 115 m), a small group of islands in the Jeju Strait off the southern coast of the Korean Peninsula. This sample represents an allopatric population of the original distribution of *P. gageodoensis* from remotely isolated southwestern islands in Korea. A voucher specimen was deposited at Sungkyunkwan University (SKK; http://115.145.139.78/bbs/board.php?bo_table=F1&wr_id=28, Seung-Chul Kim, sonchus96@skku.edu) under the voucher number SKK-210422503. A permit was not required given its collecting location and the specimen was identified by Seung-Chul Kim. Total genomic DNA was extracted from fresh leaves using the DNeasy Plant Mini Kit (Qiagen, Carlsbad, CA) and sequenced using an Illumina HiSeq 4000 sequencer (Illumina, Inc., San Diego, CA, USA) at Macrogen Corporation (Seoul, Korea). A total of 51,359,878 paired-end reads were obtained and assembled *de novo* using Velvet v. 1.2.10 (Zerbino and Birney, 2008) using multiple *k*-mers. Transfer RNAs (tRNA) and ribosomal RNAs (rRNA) were identified using ARAGORN v.1.2.36 (Laslett and Canback, 2004) and RNAmmer v.1.2 Server (Lagesen et al. 2007), respectively. Draft annotation was conducted by transferring the annotation from the plastome of *Nicotiana tabacum* (NC_001879) using Geneious R v.11.1.5 (Biomatters, Auckland, New Zealand). Gene annotation was manually corrected to match the start and stop codons and intron/exon boundaries.

The complete plastome length of *P*. *gageodoensis* (OK267273) was 156,397 bp, comprising one large single-copy (LSC; 85,768 bp), one small single-copy (SSC; 18,589 bp), and two inverted repeat (IR) regions (IR_a_ and IR_b_; 26,020 bp each). The overall GC content was 36.92%, and the plastome contained 131 genes, comprising 84 protein-coding genes with two pseudogenes (*inf*A and *ycf*1), 37 tRNA genes, and eight rRNA genes. The plastome sequence of *P*. *gageodoensis* contained 17 duplicated genes in the IR regions (seven tRNA, four rRNA, and six protein-coding genes). Fifteen genes (*ndh*A, *ndh*B, *pet*B, *pet*D, *rpl*2, *rpl*16, *rpo*C1, *rps*12, *rps*16, *trn*A-UGC, *trn*G-UCC, *trn*I-GAU, *trn*K-UUU, *trn*L-UAA, and *trn*V-UAC) contained a single intron, whereas *clp*P and *ycf*3 contained two introns. The highly conserved group II intron of the *atp*F gene was absent in *P. gageodoensis*, same as in several genera of the subfamily Rosoideae (Yang et al. 2018; Yang, Pak et al. 2019; Yang, Takayama et al. 2019; Yang et al. 2020). The 27 representative plastomes of Rosoideae were aligned with default parameters using MAFFT v.7 (Katoh and Standley, 2013), and a maximum-likelihood (ML) analysis (the best-fit model, TVM + F + I + G4) with 1,000 ultrafast bootstrap replications was performed based on the whole plastome sequences using IQ-TREE v.1.6.7 (Nguyen et al. 2015). *Rosa multiflora* (NC_039989) was used as the outgroup. The genus *Potentilla* was not monophyletic; *P. gageodoensis* was sister to four other taxa of *Potentilla*, that is, *P. freyniana*, *P. freyniana* var. *chejuensis*, *P. stolonifera*, and *P. stolonifera* var. *quelpaertensis* (100% BS, Figure 1).

**Figure 1.**
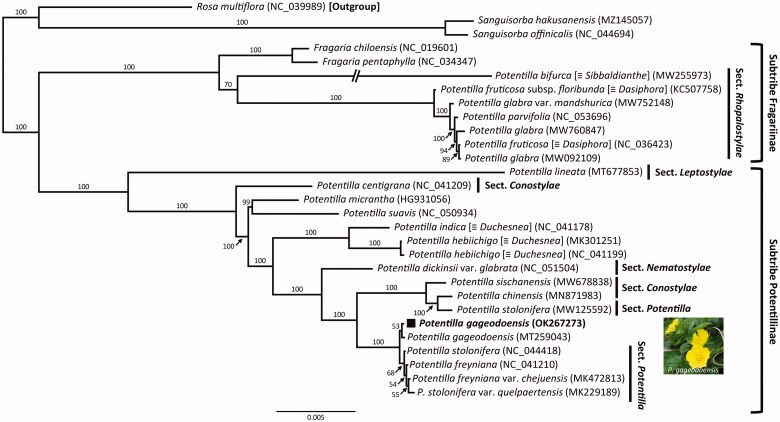
The maximum-likelihood (ML) tree based on 27 representatives of Rosoideae with the best-fit model (TVM + F + I + G4) and one outgroup taxon, *Rosa multiflora* (NC_039989). The bootstrap support value based on 1,000 replicates is shown at each node (photo credit: Seung-Chul Kim).

## Data Availability

The genome sequence data that support the findings of this study are openly available in GenBank of NCBI at https://www.ncbi.nlm.nih.gov/ (accession no. OK267273). The associated BioProject, SRA, and Bio-Sample numbers are PRJNA766785, SRR16095409, and SAMN21876083, respectively.
